# The nano-structural inhomogeneity of dynamic hydrogen bond network of TIP4P/2005 water

**DOI:** 10.1038/s41598-020-64210-1

**Published:** 2020-04-30

**Authors:** Vladimir Belosludov, Kirill Gets, Ravil Zhdanov, Valery Malinovsky, Yulia Bozhko, Rodion Belosludov, Nikolay Surovtsev, Oleg Subbotin, Yoshiyuki Kawazoe

**Affiliations:** 1 Department of Physics, Novosibirsk State University, 630090 Novosibirsk, Russia; 2 Nikolaev Institute of Inorganic Chemistry SB RAS, 630090 Novosibirsk, Russia; 3 Institute of Automation and Electrometry SB RAS, 630090 Novosibirsk, Russia; 4 Institute for Materials Research, Tohoku University, 980–8577 Sendai, Japan; 5 New Industry Creation Hatchery Center, Tohoku University, 980-8579 Sendai, Japan; 6 Department of Physics and Nanotechnology, SRM Institute of Science and Technology, 603203 Chennai, Tamil Nadu India; 7 Suranaree University of Technology, 30000 Nakhon Ratchasima, Thailand

**Keywords:** Chemical physics, Computational science, Structure of solids and liquids

## Abstract

A method for studying the time dependence of the short-range molecular order of water has been proposed. In the present study, water is considered as a dynamic network between molecules at distances not exceeding 3.2 Å. The instantaneous configurations obtained with the molecular dynamics method have been sequentially analyzed. The mutual orientation of each molecule with its neighboring molecules has been studied and the interaction energy of each pair of neighbor molecules has been calculated. The majority of mutual orientation angles between molecules lie in the interval [0°; 20°]. More than 85% of the molecular pairs in each instantaneous configuration form H-bonds and the H-bond network includes all water molecules in the temperature range 233–293 K. The number of H-bonds fluctuates near the mean value and increases with decreasing temperature, and the energy of the vast majority of such bonds is much higher than the thermal energy. The interaction energy of 80% of the H-bonding molecular pairs lies in the interval [−7; −4] kcal/mol. The interaction energy of pairs that do not satisfy the H-bond angle criterion lies in the interval [−5; 4] kcal/mol; the number of such bonds does not exceed 15% and decreases with decreasing temperature. For the first time it has been found that in each instantaneous configuration the H-bond network contains built-in nanometric structural heterogeneities formed by shorter H-bonds. The fraction of molecules involved in the structural heterogeneities increases from 40% to 60% with a temperature decrease from 293 K to 233 K. Each heterogeneity has a finite lifetime and changeable structure, but they are constantly present during the entire simulation time.

## Introduction

Water, unlike other liquids, has a disordered network of molecules connected by hydrogen bonds (H-bonds)^1^. The local structure of water is considered to be responsible for its anomalous properties^2^. Much work^3,4^ has been devoted to the search for the connection between the local structure of water and its anomalous properties.

The understanding of local structure originating from x-ray and neutron scattering data provides an idea of the time-averaged pair-distribution function of the sample. However, the structure of water evolves dynamically on a 100 fs order time scale^5–7^ and thus is widely recognized as a highly dynamic network of molecules bound by H-bonds and along with its local structure, the dynamics of the H-bonds are responsible for the unique properties of water^8^. Therefore, detailed information about the instantaneous local structure is required. This may be obtained by analyzing molecular motion using ultrafast spectroscopy as well as molecular dynamics (MD) simulation, which shows the movement of each molecule. A number of theoretical works are devoted to the description of hydrogen bonds and the study of their lifetimes^9–11^.

Ultrafast nonlinear vibrational spectroscopy has revealed that the processes of destruction and re-formation of H-bonds, as well as the vibrational motion of groups of molecules bound by H-bonds occurring on a 10 fs order time-scale^12–14^. Moreover, studies have shown the changes in the fluctuating network structure are due to collective molecular motion^13,15,16^. These results are in agreement with theoretical data^13,15–20^ and this opens the possibility for interpretation of experimental results.

In experimental studies of the structure of water, anomalous density fluctuations with dimensions of ~0.6 nm were observed^21^ and were later confirmed by theoretical investigations^22^. Subsequently, anomalous water density fluctuations have also been studied both experimentally^23–26^ and theoretically^27–32^. An increase in density fluctuations on a length-scale of 1 nm with decreasing temperature was experimentally shown for supercooled water^23^ which is consistent with recent results^24^. Based on the results of both small-angle X-ray scattering and molecular dynamics simulations, it was suggested that the density fluctuations appear as a result of stochastic processes in a one-component liquid^33^. It was demonstrated that supercooled water has mixture-like behavior of low-density and high density liquid “patches”^34^ based on calculation of the local structural index (LSI^35^). The existence of density fluctuations and the degree of their influence on the properties of water remains a current topic of discussion. As we have shown in our previous work^36^, the behavior of water viscosity near 0 °C is affected by an increase in the number of density fluctuations.

In this paper, we study the dynamic evolution of local structures formed by H-bonded and non-bonded water molecule pairs in TIP4P/2005 water. Detailed analysis is performed of the structural and energy characteristics of the time-dependent local configurations based on the mutual orientation of each pair of neighbor molecules. The study is carried out on a series of successive instantaneous configurations obtained by the MD method in the temperature range from 233 K to 293 K with a 1 fs time step at a 1 ns time-scale. In addition the existence of structural heterogeneities (SH) formed by molecules bound only by short bonds in a network of H-bonds has been studied. The structure and energy parameters of the SHs have been determined. Moreover, the time and temperature dependence of SH number, size and the fraction of involved molecules have been found. It is important to note that these heterogeneities differ from the widely studied LDL and HDL patches, where the ordering of patches within the molecular environment is investigated at oxygen-oxygen distances in two specific regions: R_OO_ < 3.2 Å and 3.2 Å < R_OO_ < 3.8 Å^37^.

## Methods

To model water, the molecular dynamics method within the approximation of a rigid molecule was used (LAMMPS software package^38^). The interaction between water molecules was described by the TIP4P/2005 potential^39^. The TIP4P/2005 water model was chosen due to its better agreement with experimental data^34,36,40^ when calculating water viscosity in the temperature range from 0 to 20 °C in comparison with other potentials, including: SPC/E, TIP4P, TIP4P/Ew^41^. Further, the average value of the water density at the selected temperature differed from the experimental data by less than 1%^39,42,43^. Finally, comparison with experiment for the isothermal compression of supercooled water also showed the most accurate results among the popular empirical potentials used for water modeling^44^.

A cubic cell consisting of 8000 molecules with periodic boundary conditions was constructed for the calculations. The molecules were evenly distributed throughout the cell: the start positions were set by the parallel transfer (copy) of one molecule with fixed orientation 20 × 20 × 20 times. This produces a highly ordered cubic supercell model with a size of 62 Å. This structure was then disordered using MD simulations over a few picoseconds^36^. The procedure for obtaining the structure of crystalline ice *I*_h_ and amorphous ice has been described previously in detail^45^.

The MD simulations were performed using an *NPT* ensemble for 1 ns with a time step of 1 fs at temperatures of 233, 253, 273, and 293 K and a pressure of 1 bar. Pressure and temperature were controlled by the Nose-Hoover thermostat and barostat^46,47^ implemented in the LAMMPS package. Long-range Coulomb interaction was calculated using the PPPM method^48^.

During the simulation, the coordinates of all atoms within the model were recorded at each instant, determining the instantaneous configurations of the system (snapshots). Within the selected time step, the motion of water molecules can be considered as continuous because a rigid water molecule does not have vibrational frequencies with a period of the order of 1 fs: the maximum typical frequency of water vibrations is about 1000 cm^−1^, and the time for one oscillation is ~33 fs. The equilibrium structures and coordinates of the water molecules were obtained and used to study the H-bond network. The average values presented in sections 1 and 2 in Results and Discussion were obtained using time interval from 250 to 1000 ps in which the equilibrated structures could be considered. Figure S1 presents the data on the potential energy during the water model equilibration and confirms the boundaries of the selected interval. To study the structural heterogeneities (SHs) in the H-bond water network, analysis of a series of snapshots has been performed.

This includes:

- determining the dependence of the distribution of the number of closest O···O pairs (R_O−O_ < 3.2 Å) on their distance, the mutual orientation angles ∠H − O···O and their interaction energy as a function of temperature;
- determining the dependence of the number of closest O···O pairs per molecule as a function of time and temperature as well as the average number of molecules in the nearest environment (short-range order);
- defining the standard H-bond formation between the O···O pairs using geometric criteria (R_O−O_ < 3.2 Å, ∠H − O···O < 30°) as well as the formation of short H-bonds (R_O−O_ < 2.76 Å, ∠H − O···O < 30°) and building the corresponding R_O−O_ and interaction energy distributions as well as the time dependence of the average number of bonds in the formation of which each molecule participates. The criteria described above were chosen due to slight differences between energy, geometric and hybrid criteria^49,50^. The R-β criterion presented in ref. ^50^ used the following parameters: R_O−O_ < 3.2 Å, β_H−O···O_ < 40°. However, as shown below the distribution of mutual orientation angles at β_H−O···O_ > 30° is insignificant;
- determining the coordinates and visualizing the molecules participating in the formation of the H-bond network and the SHs formed by short H-bonds consisting of more than 5 molecules.

The existence of an H-bond network has been investigated for snapshots obtained during 1 ns, and the study of SHs has been carried out on the data obtained during a period of 5 ps. The 5000 structures were obtained for each selected temperature and analyzed.

## Results and Discussion

### Network of bonds between molecules lying at a distance not exceeding 3.2 Å

**Figure 1:**
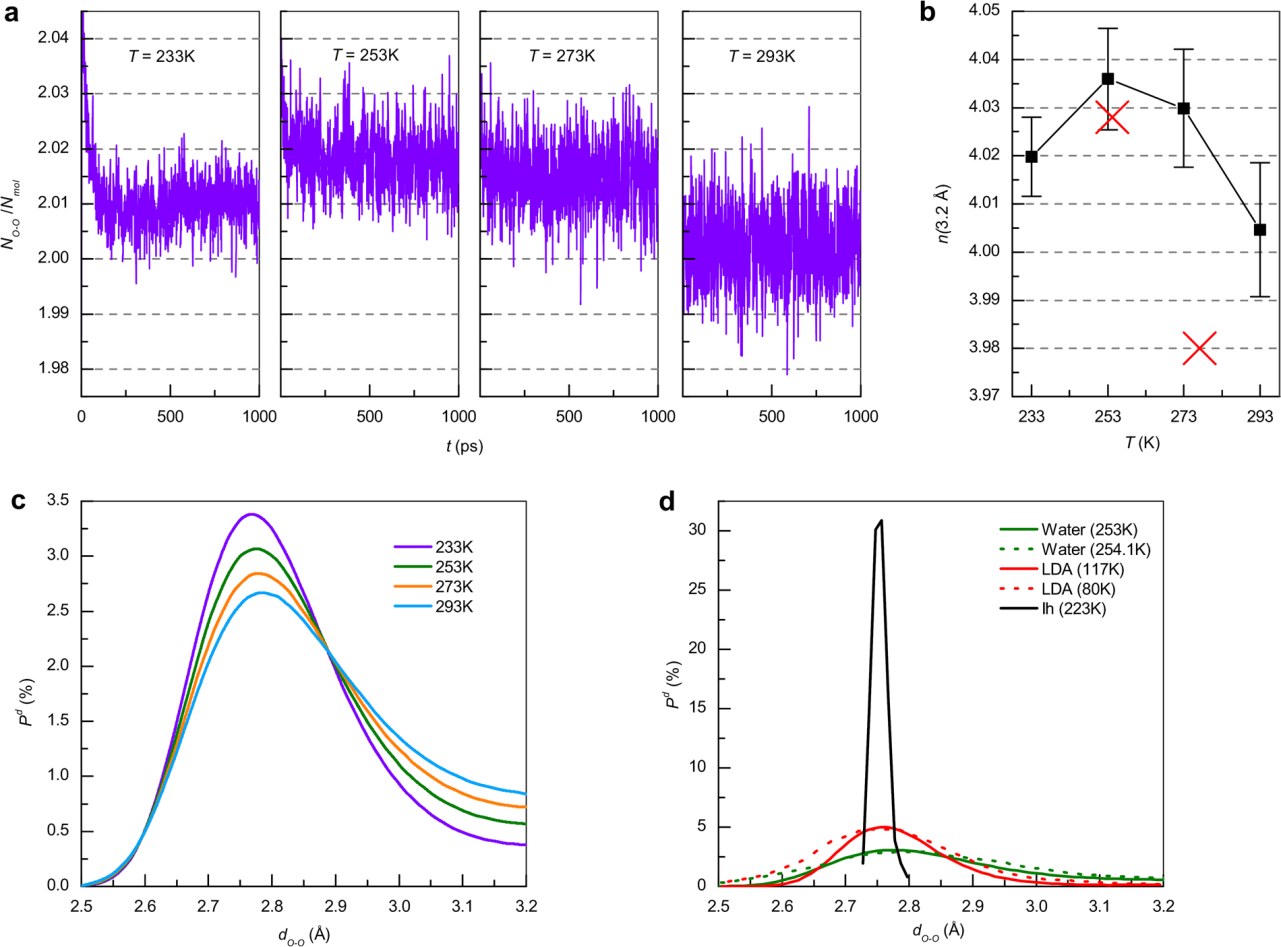
(**a**) Dependence of O···O pair number, N_O-O_, (R_O-O_ < 3.2 Å) normalized to the number of molecules N_mol_ as a function of time at 233, 253, 273 and 293 K. (**b**) Average values and standard deviations of the coordination number *n*(R_O-O_). Red crosses correspond to experimental data^51^. (**c**) Time-averaged distribution P^d^ of O···O pair lengths d_O-O_ in the water structure with a 0.01 Å step at 233, 253, 273 and 293 K and (**d**) in hexagonal crystalline (I_h_, 233 K) ice, low density amorphous (LDA, 117 K) ice and experimental data for LDA at 80 K and water at 254.1 K (dashed lines)^53^.

The proposed approach allows to one to find the time dependence of the number of pairs whose O···O distance does not exceed 3.2 Å per molecule as is shown in Fig. 1a for 233, 253, 273 and 293 K. The network of molecules bound via such pair interactions is called a bond network (BN). Figure 1b shows the temperature- and time-averaged values and standard deviations of the coordination number n(R) = 2N_O−O_/N_mol_, where N_O−O_ is the time-averaged O···O pair number and N_mol_ is the number of molecules. It can be seen from the figure that the average value of N_O−O_/N_mol_ at any temperature is ~2.01. This means that the coordination number of some molecules corresponds to more than 4 molecules. Fluctuations near the mean value of 2 mean that at a given time the coordination number of the molecules is close to 4. However, the number of such molecules, as can be seen from the magnitude of the deviation, falls with decreasing temperature. This indicates there is an increase in the number of molecules that have a coordination number of 4 molecules at a pressure of 1 atm that is natural for ice, and also indicates there is a decrease in water mobility that make the pairs more stable. The temperature correlation is an agreement with experimental results^51^. The maximum at 253 K (see Fig. 1b) corresponds to the decreasing density during the cooling of water under the 0 °C^52^.

To determine the characteristic distances of pair molecular interactions in the BN, and the dependence of these distances on temperature, the change in the time-averaged distribution of the O···O pair lengths with decreasing temperature was calculated and is shown in Fig. 1c. The peak of the distribution narrows with cooling. Figure 1d shows a comparison of the time-averaged distribution of the pair lengths in the simulation of water, crystalline ice I_h_, and low density amorphous (LDA) ice. As seen from Fig. 1d, the distribution for water at 233 K qualitatively coincides with the distribution for LDA ice. The average O···O distance between the closest molecules in crystalline ice I_h_ is ~2.76 Å. Calculated results for water at 253 K and LDA ice at 117 K agree with experimental data^53^ on the position and height of the peaks. Upon melting of crystalline ice, the smearing of pair length distribution is observed from an interval of 2.7 Å–2.8 Å to an interval of 2.5 Å–3.2 Å. The calculations show that at any time there are no molecules detached by more than 3.2 Å from any other molecule, meaning that a BN is formed by all water molecules in a select supercell and hence there are no isolated molecules or groups of molecules.

**Figure 2:**
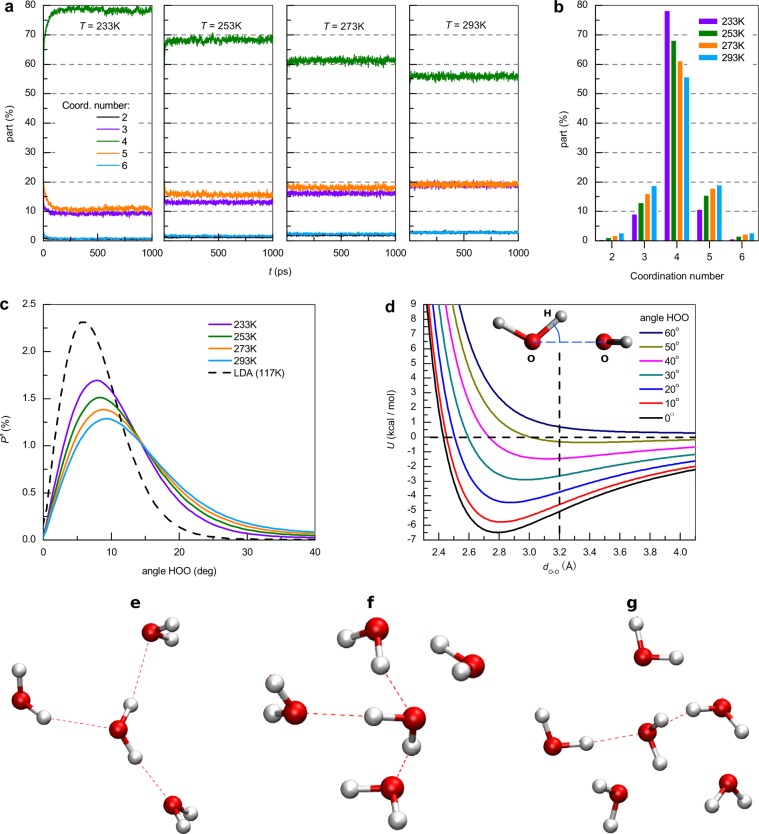
(**a**) Dependence of molecular fraction with a coordination number of 2–6 on time at 233, 253, 273 and 293 K. **(b**) Average value as a function of coordination number. (**c**) Distribution *P*^*a*^ of mutual orientation angles ∠H−O···O of closest molecules with a 1° step in water at 233, 253, 273 and 293 K and LDA ice. (**d**) Potential energy U of the two water molecule interaction as a function of O···O distance and ∠H−O···O angle. Examples of the spatial position of molecules in BN when the central molecule has (**e**) 3, (**f**) 4 and (**g**) 5 molecules in short-range order. The dashed lines represent H-bonds. This example is the result of random selection and is not the most common.

Figure 2a shows the data for the local structure (short-range order) and the distribution of the fraction of molecules in terms of their coordination number as a function of time and temperature. The number of molecules whose coordination number is 4 represents 80% of the total at 233 K and 55% at 293 K (see Fig. 2b). At the same time, the number of molecules that are surrounded by 3 or 5 molecules increases strongly with increasing temperature. This is due to the increase in the thermal activity of water molecules and the subsequent violation of the tetrahedron features of the water structure. The number of molecules whose coordination number is 2 or 6 is sufficiently small at any temperature, and for coordination numbers 1 and 7 it is insignificant (0–0.1%) and practically independent of temperature. These results are in qualitative agreement with work^54^ based on different calculation methods.

Figure 2e–g shows snapshot examples of the short-range order consisting of 3, 4 and 5 molecules (relative to the central one). It can be seen that not every pair whose O···O distance is less than 3.2 Å forms an H-bond. That is, not all pairs satisfy the angular H-bond criterion (∠H − O···O < 30°): there are 3 H-bonds in Figs. 2e,f and 2 in Fig. 2g.

It follows from Fig. 2a,b,e–g that not all neighboring molecules (satisfying the criterion R_O−O_ < 3.2 Å) within the BN form H-bonds. In view of the fact that the oxygen-oxygen distance criterion of H-bonding is satisfied for every close pair, the percentage of pairs for which the criterion for the angle of mutual orientation is satisfied must be determined. Figure 2c shows the distribution of the angles of mutual orientation of the close molecules, as well as comparison with LDA ice. The angular distribution in TIP4P/2005 water has a maximum near 8° and increases by ~ 25% with a temperature decrease from 293 K to 233 K. The overwhelming majority of angles, determined by the integral over the distribution, lies in the interval [0°; 30°], and a much smaller number lies outside this interval. TIP4P-Ew calculations^11^ confirm that violation of the O···O distance criterion does not usually happen. Therefore, N_O-O_ changes slightly with increasing temperature and violation of the angle criterion usually leads to H-bond breaking.

Figure 2d shows the result of calculation of the interaction energy (the sum of the Coulomb and Lennard-Jones interaction energies) as a function of the distance and mutual orientation angle between pairs of molecules. The potential energy has a minimum determining the equilibrium distance between molecules, and this distance essentially depends on the mutual orientation angle of the molecules. This minimum disappears at angles above 40°, and at angles less than 30° lies below −3 kcal/mol. Most of the molecule pairs (Fig. 2c) have angles less than 30°, and their interaction energy is much higher than the thermal energy (on the order of ~1 kcal/mol in the temperature range 233–293 K). The energy of the pair interaction of molecules, for which ∠H–O···O > 30°, is comparable with the thermal energy. The calculations of the interaction energy obtained using the empirical potential of the model TIP4P/2005 are qualitatively consistent with the dependencies obtained by *ab initio* methods^55^.

### Hydrogen bond network

**Figure 3:**
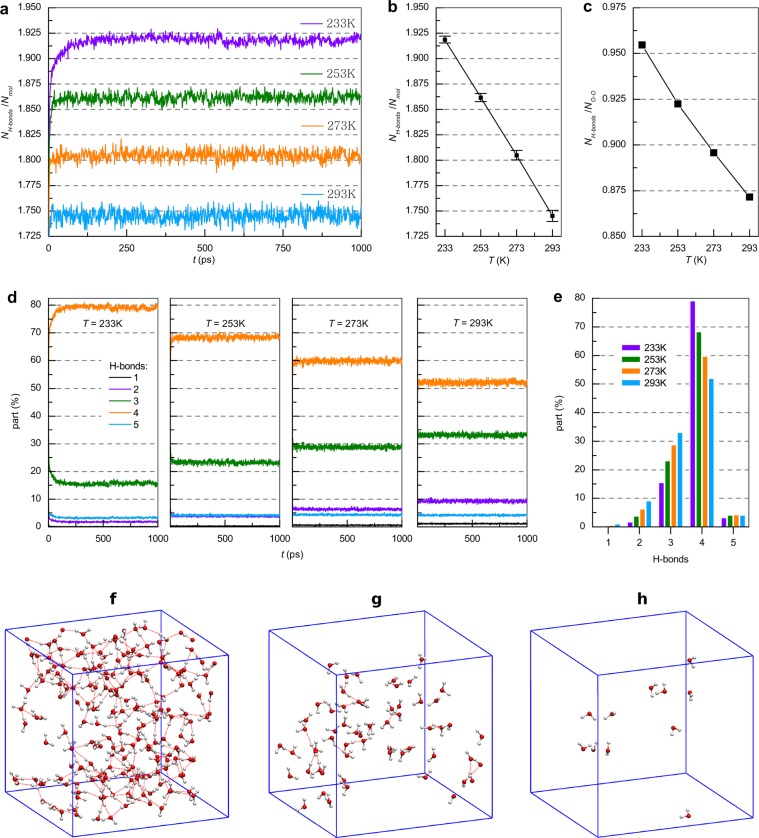
(**a**) Dependence of H-bond number N_H-bonds_ normalized to number of molecules N_mol_ as a function of time at 233, 253, 273 and 293 K. (**b**) Average values and standard deviations at the selected temperatures. (**c**) Dependence of H-bond number normalized to N_O-O_ as a function of temperature. (**d**) Dependence of molecule fraction having 1, 2, 3, 4 and 5 H-bonds as a function of time at 233, 253, 273 and 293 K. (**e**) Average values for the presented dependencies. The 20 Å × 20 Å × 20 Å region of modeling cell showing the molecules taking part in (**f**) 4, (**g**) 3 and (**h**) 2 H-bonds and H-bonds between them only.

From Fig. 2c it follows that not all BN pairs satisfy the geometric criteria of the H-bond, but the number of pairs of molecules whose mutual orientation angle ∠H−O···O is less than 30° represents the majority. Figure 3a shows the dependence of the number of pairs, for which the angular H-bond criterion is satisfied per molecule as a function of time and temperature. It can be seen that the number of H-bonds varies with time, but is near the average value, which depends on temperature. Each instantaneous configuration has a certain number of H-bonds and their number oscillates near the average value depending on the temperature and increases with decreasing temperature (Fig. 3b). In contrast to the BN, the average values of H-bond number more strongly depend on temperature and are determined by changes in the distribution of mutual orientation angles. More than 85% of the BN pairs form H-bonds at given temperatures; as seen in Fig. 3c the average value differs from 2 by ~4% at 233 K, by ~8% at 253 K, by ~11% at 273 K, and by ~% at 293 K, which corresponds to the results obtained by Pauling^56^. This agrees qualitatively with TIP4P-Ew calculations with a slightly larger O-O distance criteria^11^. The results shown in Fig. 3b indicate that on average each molecule participates in at least 3.5 H-bonds at a given temperature. Analysis of the instantaneous structures shows that at any time the entire system (as in the BN, all water molecules) forms a single and long-range network of H-bonds and based on Fig. 2c,d one can draw a conclusion about the energy stability of the H-bond network. As the temperature rises, some bonds break, but a unified H-bond network remains that could be named an extended H-bond network, as suggested by a recent theoretical study^19^. This means that isolated chains, clusters or heterogeneities cannot exist outside the H-bond network, but they can be built into the network.

To determine the short-range order of H-bonded molecules, the time and temperature dependencies of the fraction of molecules participating in 1, 2, 3, 4, and 5 H-bonds were obtained. Figure 3d shows that the number of molecules forming 4 H-bonds decreases with increasing temperature, and the number of molecules forming 1, 2, and 3 bonds increases. Thus, at a low temperature the potential energy of the molecules is higher (in absolute value) due to the greater number of strong bonds and number of molecules forming 4 H-bonds, which indicates an increase in the strength of the H-bond network with decreasing temperature. The results presented at Fig. 3d are in good agreement with previous results^57^. At each temperature, oscillations occur near the mean value given in Fig. 3e. Most molecules participate in 3 and 4 H-bonds, which is not in agreement with the experimentally obtained values of 2.2 ± 0.5^26,58^. This may be related to limitations of the TIP4P/2005 model with fixed-charged molecules, which is a simplification of real water. However, the TIP4P/2005 model has been used to describe selected water properties with reasonable accuracy^39,59^. These results distinguish the H-bond network from the BN, where the number of molecules having a coordination number of 5 is comparable to the number of molecules having 3 molecules in the short-range environment (Fig. 2b).

It can be seen from Fig. 3e that most of the molecules participate in 4 H-bonds: 78% at T = 233 K; 68% at 253 K; 59% at 273 K; 51% at 293 K. The fraction of molecules participating in 5 H-bonds does not exceed 5% and slightly increases with increasing temperature. The fraction of molecules participating in 3 H-bonds increases with increasing temperature from 15% to 33%, and the fraction of molecules participating in 2 bonds increases from 2% to 9%. The number of molecules forming 1 H-bond is insignificant. These results agree well with early ST2 simulation data and predictions provided by Stanley *et al*.^60^. Thus, water in the supercooled state has a greater number of H-bonds, as well as a larger number of molecules involved in 4 bonds.

Figure 3f represents the small region (20 Å × 20 Å × 20 Å) of the instantaneous spatial arrangement of only those molecules that participate in 4 H-bonds, as well as the H-bonds formed between them at 253 K. This example is typical for any time and temperature. Figures 3g,h show the arrangement of only those molecules that participate in 3 and 2 bonds, respectively. The figure shows that the molecules involved in 4 H-bonds form a network of H-bonds, and molecules that have a smaller number of bonds are localized and almost unconnected by H-bonds to each other.

Based on the calculated spatial distribution, it can be concluded that the water molecules are divided into two groups. The first group comprises the molecules that make up the basis (~60–80%) of the H-bond network and participate in 4 or more H-bonds. This group forms a long-range network. The second group consists of molecules participating in 3 or fewer H-bonds, the number of which is much smaller.

**Figure 4:**
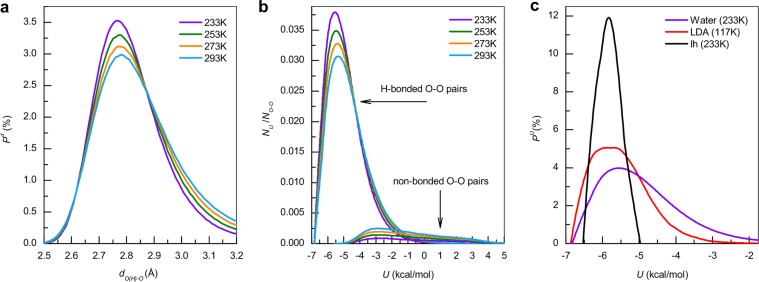
(**a**) Distribution of H-bond lengths P^d^ with 0.01 Å step at 233, 253, 273 and 293 K. Time-averaged distribution of pair potential energy N_U_ (R_O−O_ < 3, 2 Å) normalized by the average number of pairs N_O−O_ for H-bonded and non-bonded pairs (**b**) of TIP4P/2005 water at 233, 253, 273 and 293 K and (**c**) Ih ice and LDA ice with step 0.1 kcal/mol.

Figure 4a shows the distribution of H-bond lengths in TIP4P/2005 water at temperatures of 233, 253, 273 and 293 K. The main peak lies in the interval [2.7; 2.9] Å and grows by 25% with decreasing temperature. The main difference between these results and the above-mentioned BN distributions (see Fig. 1c) is the lower number of O···O pairs, the distance between which lies in the interval [3.0; 3.2] Å. Considering that the most of the angles of mutual orientation lie in the interval [0°; 30°] (see Fig. 2c), it can be concluded that the network is formed by H-bonds with a small spread of lengths and angles of mutual orientation. According to Fig. 2d such bonds have the highest energies (in absolute value). This may indicate the stability of the H-bond network, but a more detailed study of the energy for all pair interactions is required.

The energetic (thermodynamic) properties of the H-bond network are determined both by strong (significantly exceeding the thermal energy of ~0.6 kcal/mol) and weak (comparable to the thermal energy) pair interactions. Figure 4b shows the time-averaged distributions of the potential energy of H-bonded and unbounded pairs (R_O−O_ < 3.2 Å) normalized by the average number of pairs for the representative temperature. The energy of the pair interaction was calculated in the same way as in Fig. 2d. The position of the maxima lies in the region of [−5.5; −5] kcal/mol and slightly depends on the temperature. The energy of most H-bonds lies in the interval [−7; −3] kcal/mol. This interval determines the stability of H-bond network. The growth of the peak with decreasing temperature is due to the increase in the number of H-bonds. It can be seen that there are H-bonds that have energy [−1; 2] kcal/mol. This is due to the low intermolecular distance and, accordingly, the mutual repulsion of the molecules. The energy distributions of pairs without H-bonds are uniformly distributed in the interval [−5; 5] kcal/mol. At the same time, the values are significantly less than for H-bonded pairs, and are shifted to a region of positive energy. The weakly bonded molecules do not have a coordination of 4 and this leads to structural reorganization^54,61^.

Figure 4c shows the energy distributions of the pair interaction energy in crystalline ice Ih and LDA ice. The values lie in the interval [−7; −5] kcal/mol that determines the stability of these structures. From this figure it can be seen that amorphous and crystalline ices have a narrower range of potential energies. This means that each molecule in I_h_ and LDA ices oscillates near the equilibrium position determined by strong interactions with the nearest molecules. According to Figs. 1d and 2d ∠H−O···O in ice I_h_ can vary in the interval [0°; 10°]. Temperature also affects the liquid water structure consisting of molecules bound by strong H-bonds, but a more significant effect is on molecules bound by weak bonds that can lead to structure reorganization. This fundamentally distinguishes water from the solid phases.

### Nano-scale structural heterogeneities in the dynamic hydrogen bond network

As was shown in the previous section, there are no isolated molecules or groups of molecules that can form clusters. There remains the question of the existence of the SHs formed by molecules bound only by short bonds in a network of H-bonds. For this purpose, the number of molecules bound by short H-bonds was calculated and their coordinates were found for 5000 snapshots, which correspond to a 5 ps simulation time for the considered temperatures. The number of pairs that do not satisfy the angle criterion, i.e. have R_O···O_ < 2.76 Å and ∠H − O···O > 30°, is extremely small and are therefore not counted. This is explained by the strong repulsion of negatively charged oxygen ions (Fig. 2d).

**Figure 5:**
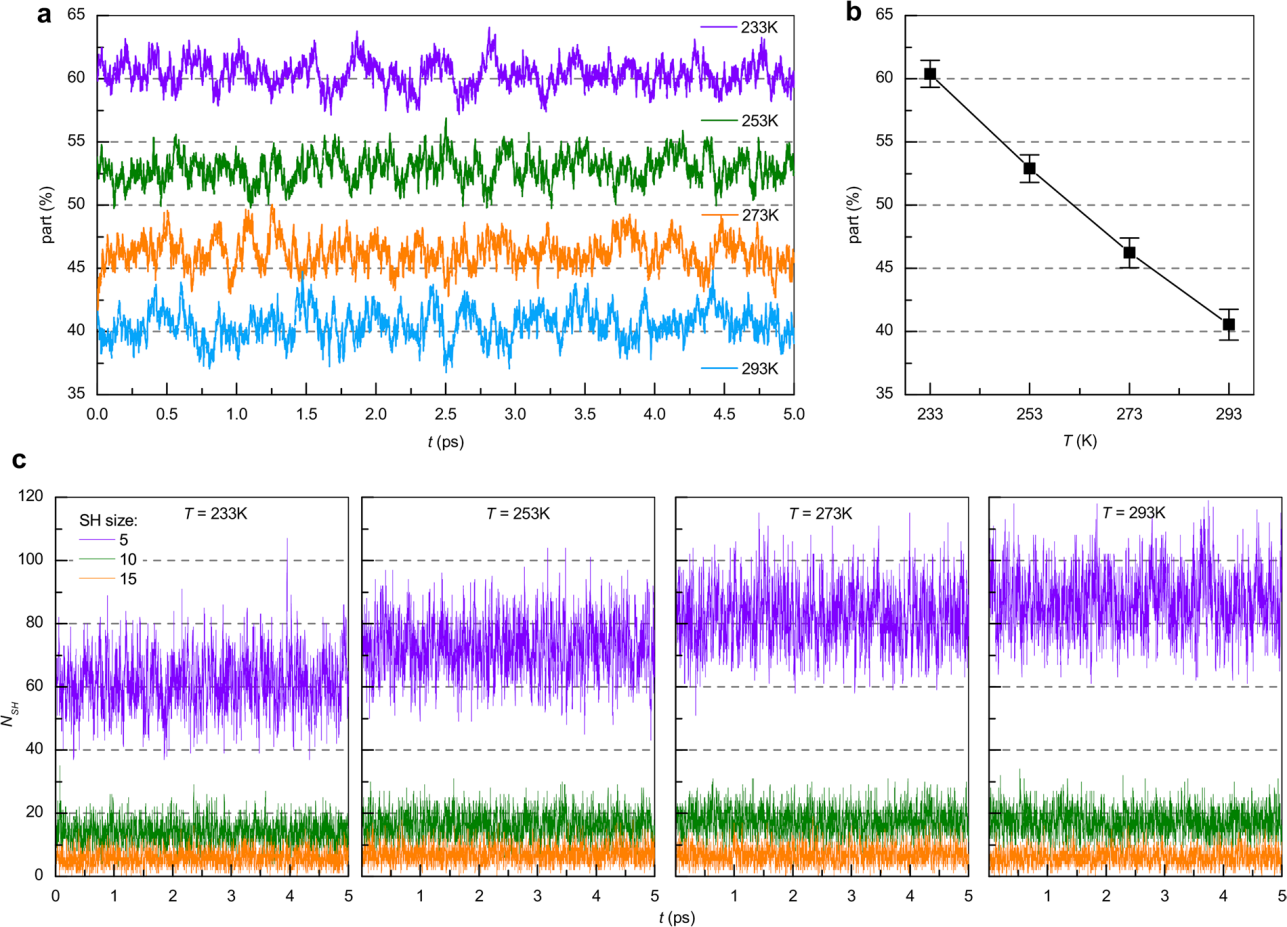
(**a**) Time dynamics of the molecular fraction forming SHs at 233, 253, 273 and 293 K. (**b**) Average values and standard deviations for the temporal dependencies. (**c**) Dependence of SH number N_SH_ consisting of 5, 10 and 15 molecules on time at 233, 253, 273 and 293 K.

Figure 5a shows the dependence of the percentage of molecules forming SHs, which consist of 5 or more water molecules, on temperature and modeling time. As the temperature is lowered, the average number of SH forming molecules increases (see Fig. 5b). Despite strong binding between the molecules, their number fluctuates strongly: SHs form and decay over the course of the simulation time. The average value for 233, 253, 273 and 293 K are 61%, 53%, 46% and 41%, respectively. This means that it is impossible to form a long-range network of short H-bonds. The standard deviation decreases with decreasing temperature from 293 K to 233 K. As for the BN pairs, there is a decrease in the molecular mobility and therefore a weakening of the temperature influence on the water structure.

The SHs discussed here are groups of molecules bound by short H-bonds, which have different sizes and are distributed throughout the volume of the model cell.

Figure 5c shows the time dependence of the number of SHs consisting of 5, 10 and 15 water molecules at temperatures of 233, 253, 273 and 293 K (see the supplementary materials for video files showing the spatial distribution of 5, 10, 15 and 20 molecule size SHs with 1 fs time step). It can be seen that these numbers fluctuate around the mean value. SHs of size equal to 5 are always present, but their number is not constant and fluctuates in the range of 40 to 80 heterogeneities. Similar qualitative results were obtained for SHs of sizes 10 and 15. The time-averaged number of SHs of a certain size for different temperatures is given in Table 1. This was calculated by the following formula: $\frac{1}{n}\mathop{\sum }\limits_{{\rm{t}}=1}^{n}{N}_{S}(t)$, where *n* is the number of simulation time steps equal to 5000, and *N*_*S*_*(t)* is the number of SHs of size *S* at time *t*. At 233 K the number of small SHs is lower than at higher temperatures, but the number of large SHs containing more than 40 molecules is much higher, which provides a greater number of molecules involved in the heterogeneities. At higher temperatures, large SHs decay quickly, so that their average number is smaller than at lower temperatures.

**Table 1:** Time-averaged number of SHs of given size S at 233, 253, 273 and 293 К.

*S*\*T*	233 K	253 K	273 K	293 K
5	61.27	72.63	82.19	87.19
8	21.86	26.51	29.09	29.83
10	13.83	16.27	17.4	17.21
15	5.79	6.94	6.74	6.07
20	3.08	3.58	3.4	2.68
25	1.89	2.19	1.81	1.37
30	1.3	1.37	1.11	0.76
40	0.71	0.71	0.47	0.25
50	0.44	0.36	0.2	0.09
75	0.18	0.11	0.03	0.01
100	0.08	0.04	0.008	0.003

**Figure 6:**
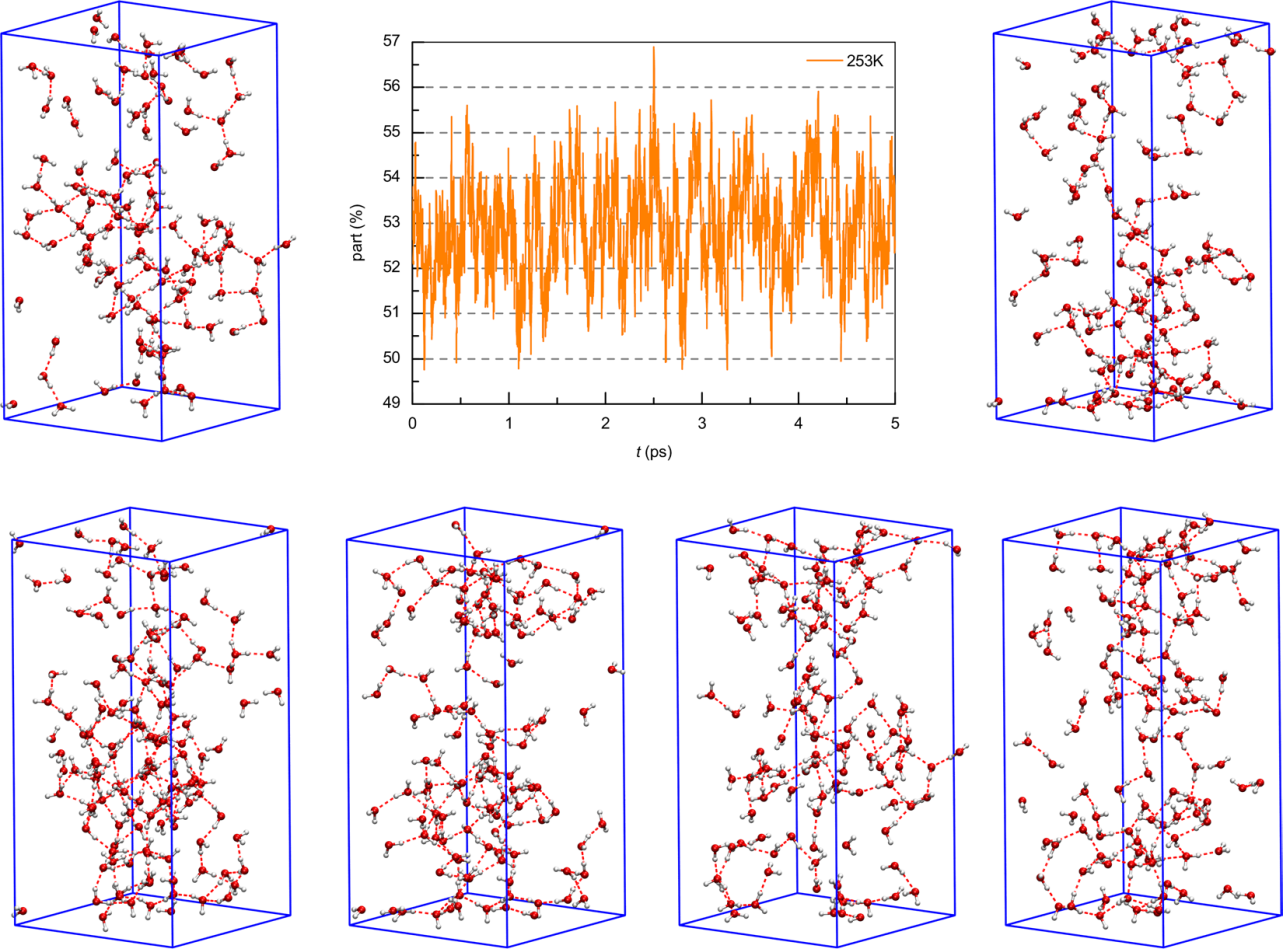
Spatial distribution of SHs containing more than 5 molecules, and the short H-bonds between them at 253 K with an interval of 1000 fs (small regions are shown for clarity). Molecules that do not participate in the formation of SHs and the connections between them are not shown. Single molecules belonging to SHs but lying outside the region are also shown.

For clarity, Fig. 6 represents the small regions of simulated cells and shows the SHs, which contain more than 5 water molecules, and short H-bonds between their molecules with 1 ps time step at 253 K. The SHs are located throughout the whole volume, and their number and size grow with decreasing temperature; however, over time these values make small fluctuations in accordance with the results shown in Fig. 5a. The SHs were found to have nanometer size, which agrees with previous results^23^.

**Figure 7:**
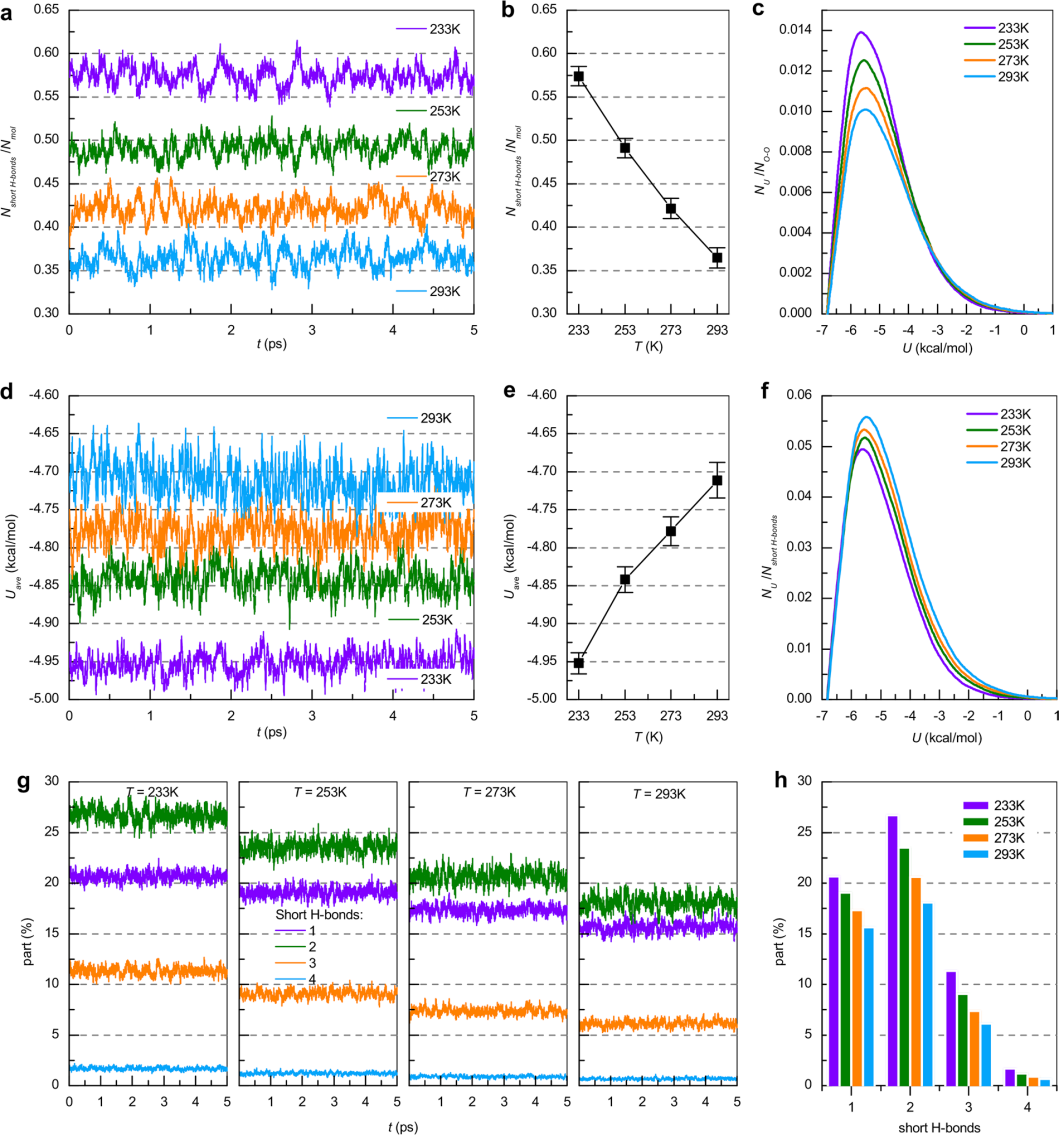
(**a**) Dependence of short H-bond number N_short H-bonds_ normalized to molecule number N_mol_ as a function of time at 233, 253, 273 and 293 K. **(b**) Average values and standard deviation for the presented dependencies. (**c**) Time-averaged distribution of forming short H-bond pair potential energy N_U_ normalized by the average value of N_O−O_ of water at 233, 253, 273 and 293 K with step 0.1 kcal/mol. (**d**) Time dependence of average potential energy *U*_*ave*_ of short H-bonds in SHs and (**e**) time-averaged values with standard deviation. (**f**) Time-averaged distribution of forming short H-bond pair potential energy N_U_ normalized by the average value of N_short H-bonds_ at 233, 253, 273 and 293 K with step 0.1 kcal/mol. (**g**) Dependence of molecular fraction taking part in 1, 2, 3 and 4 short H-bonds as a function of time at 233, 253, 273 and 293 K. (**h**) Average values for the presented dependencies.

Figure 7a,b show the time and temperature dependencies of the number of short H-bonds that bind the water molecules in the SHs (short H-bonds, which bind 2, 3 or 4 water molecules are not counted), as well as the mean time value with the standard deviation. On average, each molecule in the SH participates in 1.14 short H-bonds at 233 K, 0.97 short bonds at 253 K, 0.84 short bonds at 273 K, and 0.74 short bonds at 293 K. The number of short H-bonds increases with decreasing temperature, which confirms the growth of the size and number of large SHs (see Table 1).

Figure 7c shows the time-averaged distribution of pair potential energy between molecules that form short H-bonds in SHs normalized to the total number of O···O pairs (R_O−O_ < 3.2 Å). Qualitatively, these results agree with the distribution of the H-bond network (Fig. 4b): the peak center is at ~ −5.5 kcal/mol and lies in the interval [−7; −3] kcal/mol. It is also possible to see the peak growth with temperature decrease. Figure 7f shows a similar distribution normalized to the average number of short H-bonds, in which the energy of SHs depends weakly on temperature. The time dependence of the average value of the short bond energy that connects the molecules in the SH, as well as the time-averaged value, are shown in Fig. 7d,e, respectively. It can be seen that the standard deviation greatly increases with increasing temperature: the most energetically favorable position for a water molecule corresponds to the highest boundary value of the short H-bond O···O distance (see Fig. 2d) so increasing mobility leads to broad energy fluctuations.

Figure 7g shows the temperature and time dependencies of the percent of SH molecules participating in 1, 2, 3 and 4 short H-bonds, in which the fraction of molecules participating in 1, 2, 3 and 4 short bonds grows with decreasing temperature. At each temperature, oscillations occur near the mean value given in Fig. 7h. The proportion of molecules participating in 1 and 2 short H-bonds is comparable. At the same time, the fraction of molecules forming 1 short H-bond is less, which leads to the conclusion that the SHs are spatially localized. The fraction of molecules involved in 3 and 4 short H-bonds is small, which allows us to conclude that the structure of the SHs are “one-dimensional” – chains of molecules connected by short H-bonds.

The total percentage of molecules forming 2, 3, and 4 short H-bonds at T = 233 K is ~39.5%, at T = 253 K is ~34%, at T = 273 K is ~28.5%, and at T = 293 K is ~24%. The existence of these molecules causes the formation of nanometer SHs consisting of more than 5 water molecules, as presented in Table 1.

To show the permanent presence of structural heterogeneities in the H-bond network, additional simulations were performed over 1 ns instead of 5 ps. The results of these calculations are given in Supplementary Materials and show that deviations from the average value of the number of molecules forming heterogeneities (Fig. S2 in Supplementary Materials) consisting of 5 or more molecules are preserved and do not exceed the value for 5 ps, which confirms the constant presence of structural heterogeneities at different temperatures. By analogy, the normalized number of short H-bonds (Fig. S3 in Supplementary Materials) agrees with the 5 ps data.

It is natural to expect that the cooperative movement of molecules in heterogeneities changes the relaxation times in water. This means that molecules connected by short H-bonds have a higher degree of cooperation in relaxation processes, which increases the activation barrier and leads to a change in the behavior of the water viscosity near 0° C from the Arrhenius law, described by thermo-activation, to the non-Arrhenius law.

## Conclusions

Studying the time dependence of the short-range order of each molecule reveals the existence of a dynamic H-bond network characterized by energetic properties and configurations that change throughout the simulation. Within the TIP4P/2005 model, it was shown that the network is formed by more than 85% pairs of neighbor molecules. The number of H-bonds fluctuates with time near the average value that decreases with increasing temperature due to molecular rotation (violation of angular H-bond criterion).

The calculations showed that the interaction energy of most neighboring molecules forming an H-bond greatly exceeds the thermal energy and defines the stability of the H-bond network that is responsible for water’s properties. The interaction energy of the rest of the hydrogen bound pairs and non-hydrogen bound pairs is comparable to the thermal energy and determines the possibility of local network structure reorganization being responsible for the liquid behavior of water. In fact, the bond type is determined by the mutual orientation of neighboring molecules (the short-range order of each molecule) and can vary with time. Therefore, the network of bonds can simultaneously affect the properties of solid and the properties of liquid.

The calculations (Fig. 2a) show the coexistence of a group of molecules with a coordination number of 4 (57–80%) and a coordination number of 5 (20–11%), which indicates the possibility of the coexistence of the two actively studied phases of LDL and HDL. At a pressure of 1 bar and with decreasing temperature, the number of molecules with a coordination number equal to 4 increases from 57% to 80% of the total number of molecules, and the number of molecules with a coordination number equal to 5 falls from 20% to 11%. These groups can take part in the formation of the LDL and HDL phases; such an analysis has not been conducted in the present work, but is planned for future study.

Nano-scale structural heterogeneities that are embedded in the H-bond network were discovered for the first time. The size, quantity and total fraction of involved molecules are temperature dependent, and the spatial position changes with time. Structural heterogeneities have finite lifetimes, but accounting for their constant presence and the high percentage of the molecules that form them is important to describing the macroscopic properties of water.

## Supplementary information

Supplementary Materials.

Supplementary Video 1.

Supplementary Video 2.

Supplementary Video 3.

Supplementary Video 4.
