# Supplementary material for: The nano-structural inhomogeneity of dynamic hydrogen bond network of TIP4P/2005 water
Source: Sci Rep. 2020 Apr 30;10:7323. doi: 10.1038/s41598-020-64210-1 (PMC7192952; doi:10.1038/s41598-020-64210-1)
Supplement: Supplementary file 1 — Supplementary Materials. [file 41598_2020_64210_MOESM1_ESM.doc]

**Supplementary materials**

The nano-structural inhomogeneity of dynamic hydrogen bond network of TIP4P/2005 water

Vladimir Belosludov, Kirill Gets, Ravil Zhdanov, Valery Malinovsky, Yulia Bozhko,
Rodion Belosludov, Nikolay Surovtsev, Oleg Subbotin, Yoshiyuki Kawazoe

**1. The potential energy of TIP4P/2005 water.**

The potential energy of TIP4P/2005 water fluctuates around the average value after 250 ps of simulation for all temperature values. It confirms that in the [250; 1000] ps interval the system is equilibrated and can be used for fluctuation analysis.


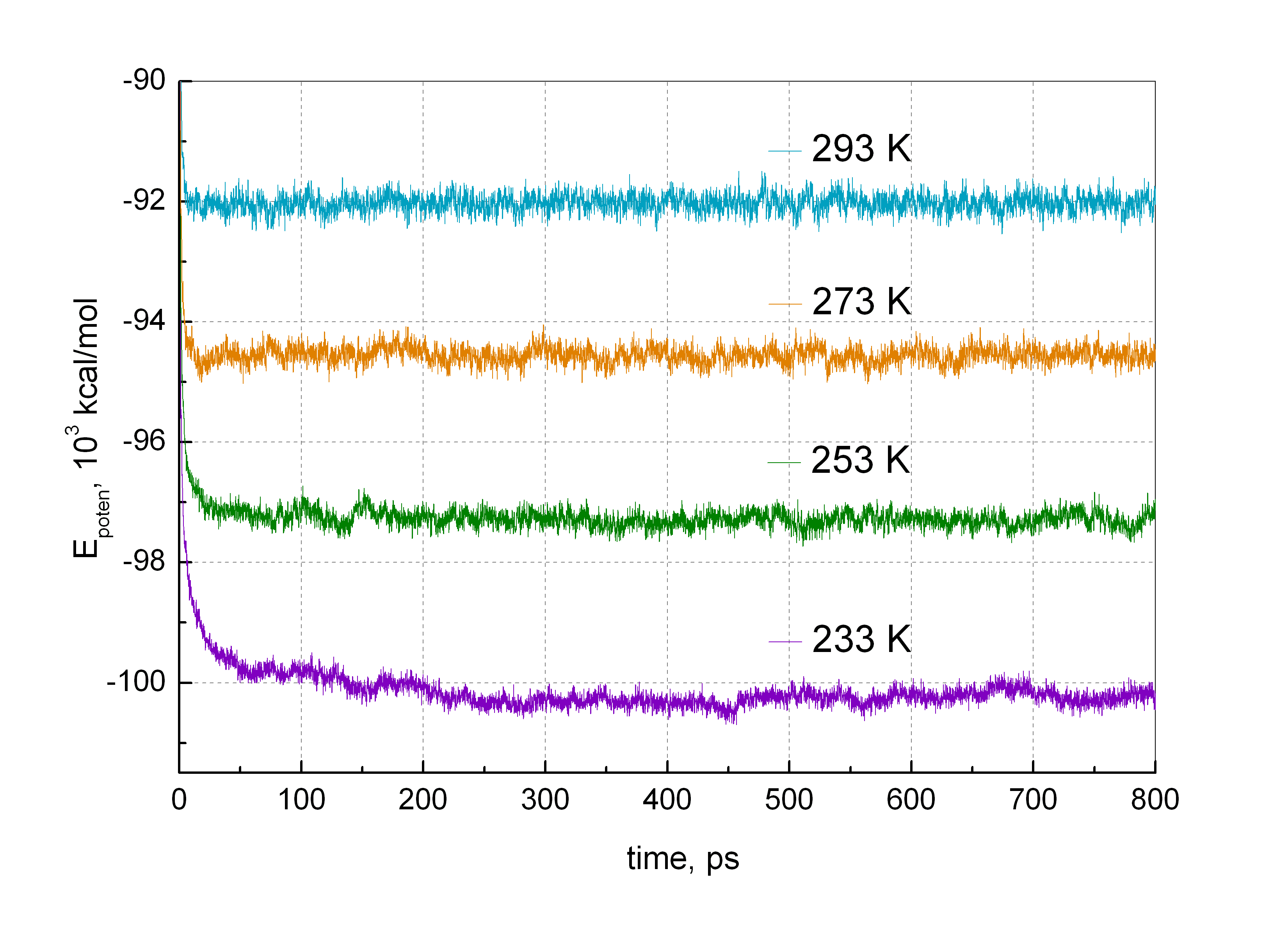


**Figure S1.** Time dependence of potential energy for TIP4P/2005 water at 293, 273, 253 and 233 K.

**2. Molecular fraction forming structural heterogeneities and number of short H-bonds.**

Time dependencies over 1000 ps of molecular fractions forming structural heterogeneities (SHs) (**Fig. S2**) and normalized number of short H-bonds (**Fig. S3**) show the same behavior as results obtained within a period of 5 ps. The average values and standard deviations obtained during 1000 ps and 5 ps are in agreement. This confirms the constant presence of SHs in the H-bond network structure in normal and supercooled states.


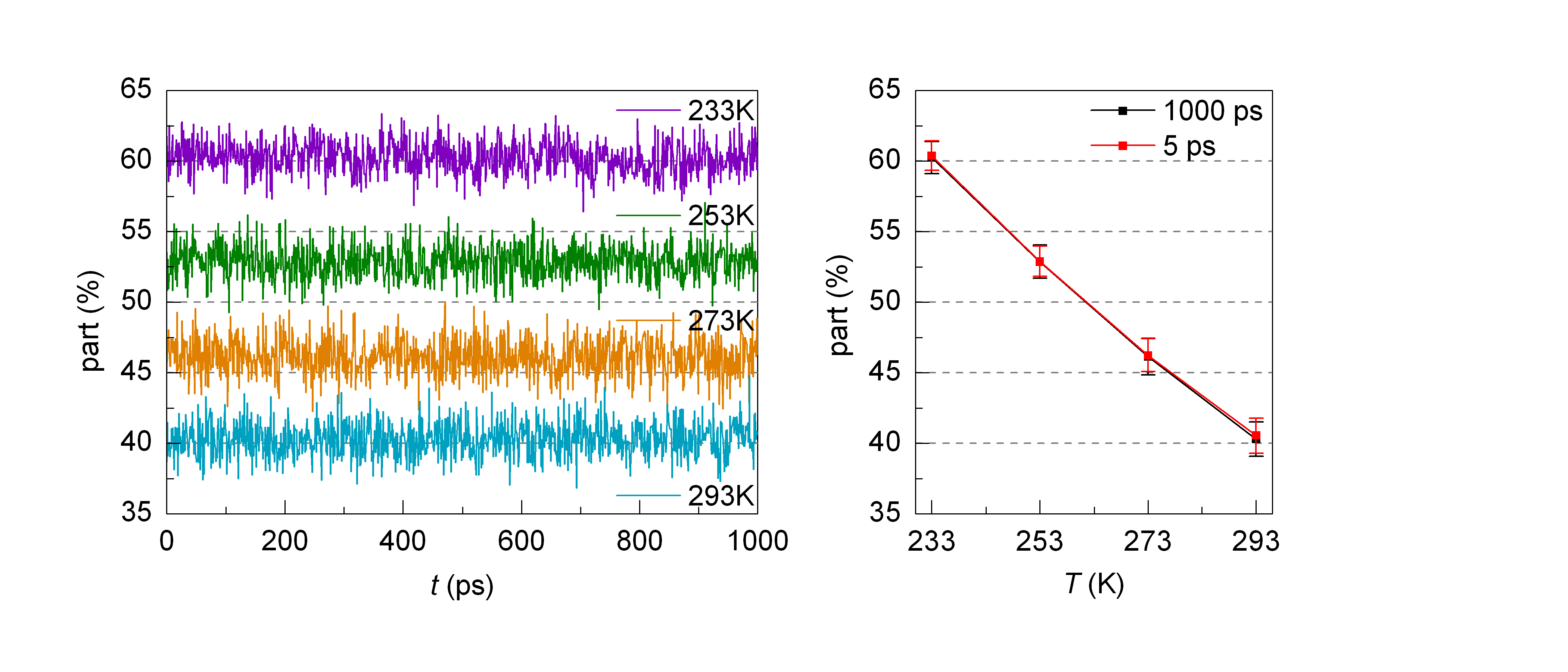


**Figure S2.** (left) Time dynamics over 1000 ps of the molecular fraction forming 5 SHs at 233, 253, 273 and 293 K. (right) Comparison of the average values and standard deviations for the temporal dependencies over the 1000 ps and 5 ps simulation times.


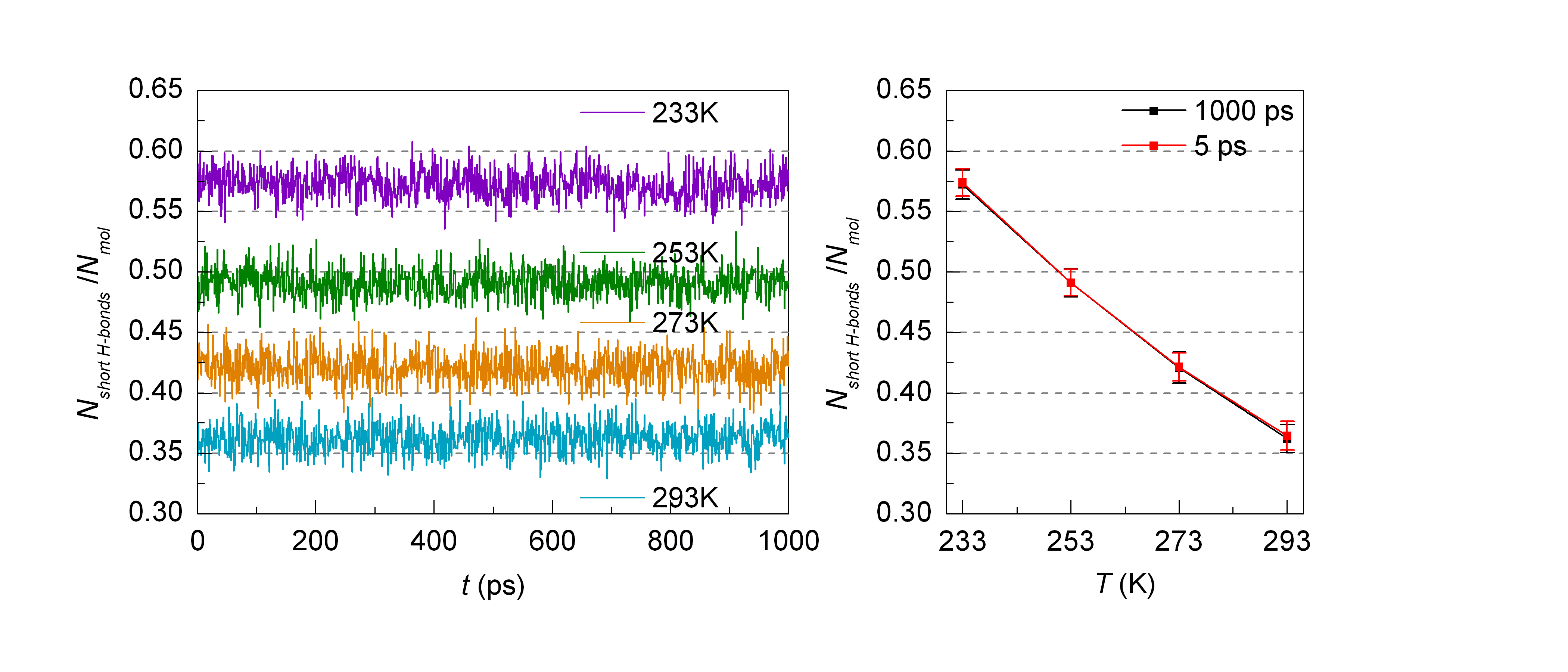


**Figure S3.** (left) Dependence of short H-bond number *N_short H-bonds_* normalized to number of molecules *N_mol_* as a function of time (1000 ps) at 233, 253, 273 and 293 K. (right) Comparison of the average values and standard deviation for the presented dependencies for 1000 ps and 5 ps simulation times.
