# Supplementary figures and images for: The nano-structural inhomogeneity of dynamic hydrogen bond network of TIP4P/2005 water
Source: Sci Rep. 2020 Apr 30;10:7323. doi: 10.1038/s41598-020-64210-1 (PMC7192952; doi:10.1038/s41598-020-64210-1)

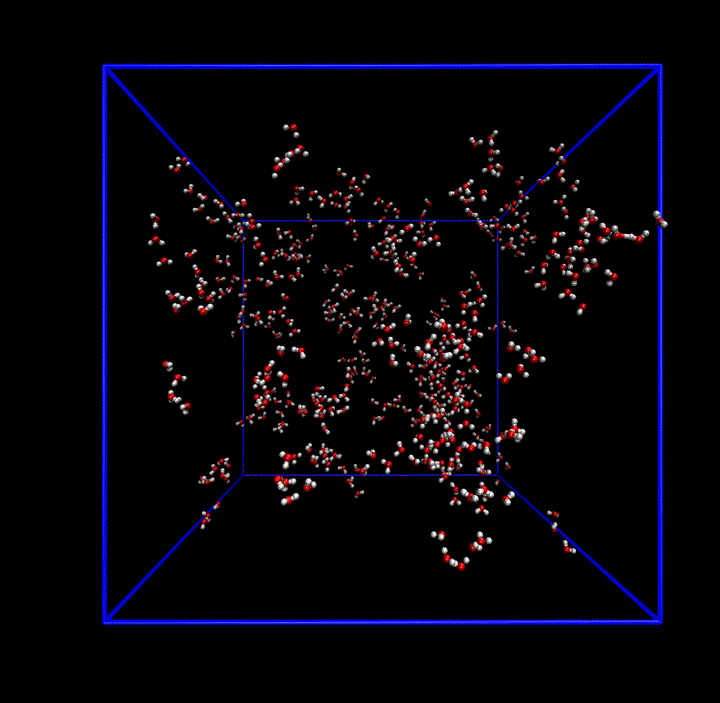

Supplement: Supplementary file 2 — Supplementary Video 1. [file 41598_2020_64210_MOESM2_ESM.gif]

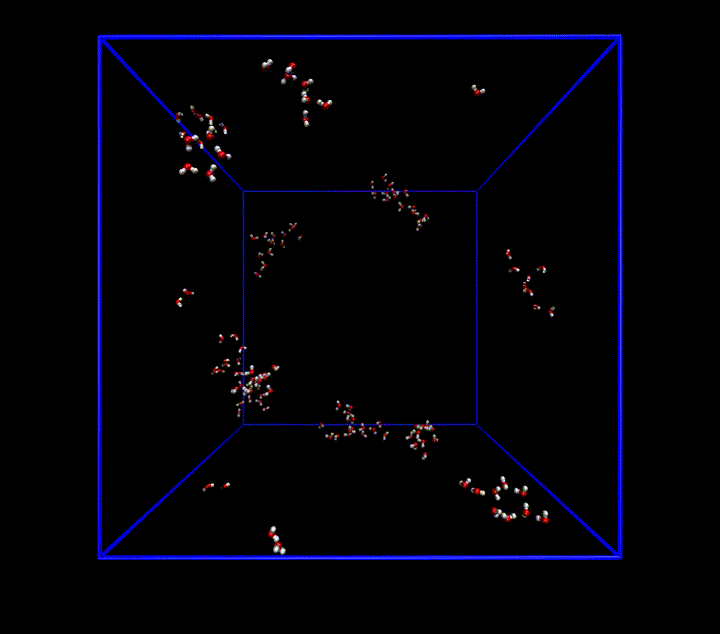

Supplement: Supplementary file 3 — Supplementary Video 2. [file 41598_2020_64210_MOESM3_ESM.gif]

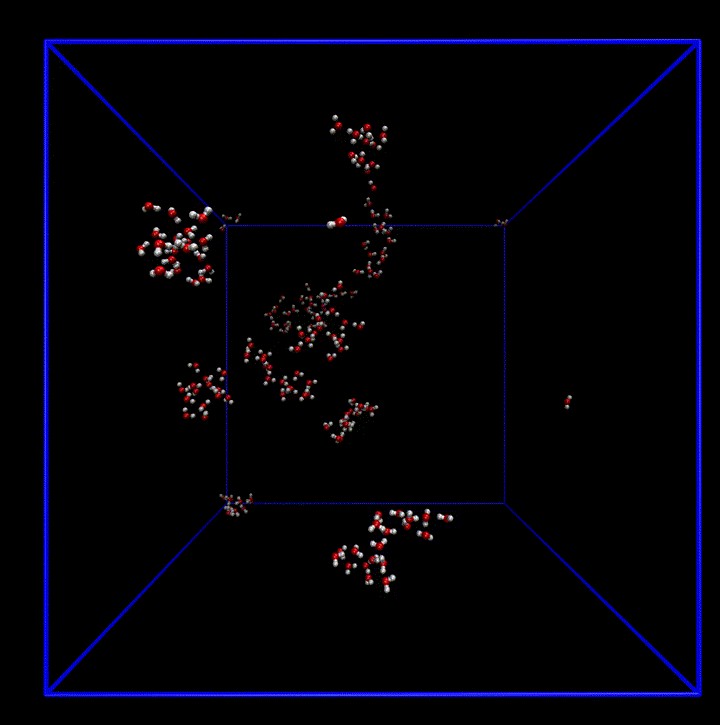

Supplement: Supplementary file 4 — Supplementary Video 3. [file 41598_2020_64210_MOESM4_ESM.gif]

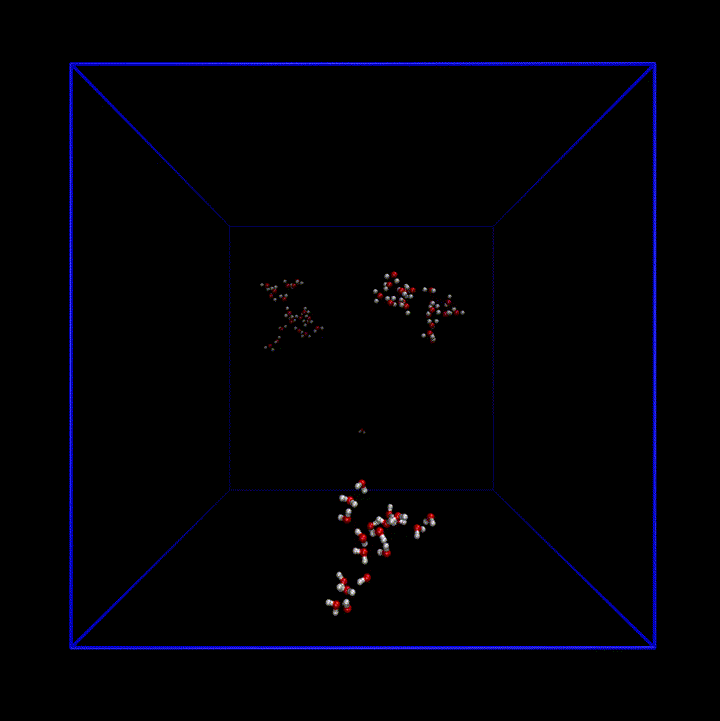

Supplement: Supplementary file 5 — Supplementary Video 4. [file 41598_2020_64210_MOESM5_ESM.gif]
